# Epigallocatechin-3-Gallate: A potential amyloid Fibril Disaggregator of Serum amyloid A1

**DOI:** 10.1016/j.bbrep.2025.102365

**Published:** 2025-11-16

**Authors:** Natalie G. Horgan, Anabela Djurovic-topalovic, Taiwo A. Ademoye, Hannah I. Reyes-Charles, Natsumi Kobayashi, Germán Plascencia-Villa, George Perry, Tomoaki Murakami, Jessica S. Fortin

**Affiliations:** a Basic Medical Sciences, College of Veterinary Medicine, Purdue University, 625 Harrison Street, West Lafayette, IN, 47907, USA; b Laboratory of Veterinary Toxicology, Tokyo University of Agriculture and Technology, 3-5-8 Saiwai-cho, Fuchu, Tokyo, Japan; c Department of Neuroscience, Developmental and Regenerative Biology, The University of Texas at San Antonio, San Antonio, TX, 78249, USA

**Keywords:** Amyloid A (AA), AA amyloidosis, Dynamic light scattering (DLS), Epigallocatechin-3-gallate (EGCG), Serum amyloid A1 (SAA1), Transmission electron microscopy (TEM)

## Abstract

Serum amyloid A1 (SAA1) is a 122-amino acid protein that, after cleavage, matures into a 104-amino acid form. Its N-terminus is responsible for binding high-density lipoprotein (HDL), while the C-terminus maintains its structural integrity. As an acute-phase protein, SAA1 is produced by the liver in response to acute inflammation. SAA1 is also a precursor to amyloid A (AA), and its accumulation can lead to AA amyloidosis—a condition secondary to chronic inflammation that causes tissue damage and organ dysfunction. Our study explores methods to disaggregate SAA1 fibrils isolated from the cat spleen, chicken liver, and cow liver. Specifically, we investigate the use of epigallocatechin-3-gallate (EGCG), a polyphenolic flavonoid extracted from green tea known for its anti-inflammatory and antioxidant properties, to disaggregate these fibrils. Dynamic light scattering (DLS) and transmission electron microscopy (TEM) were used to analyze these fibrils after treatment with 1 % DMSO and 400 μM of EGCG in 10 mM PBS (pH 7.4). The results demonstrated that EGCG effectively reduced fibril size, as confirmed by DLS characterization, with the disappearance or diminished prominence of the 10^3−4^ nm peak. Additional TEM results confirmed that EGCG disaggregated amyloid-beta fibrils isolated from Alzheimer's disease brains. These findings suggest that compounds like EGCG could be valuable in treating inflammatory and neurodegenerative conditions by disaggregating amyloid fibrils.

## Highlights

• Reduces the size of SAA1 aggregates
• Breaks down SAA1 fibrils
• Disaggregates Aβ fibrils

## Graphical abstract

**Figure:**
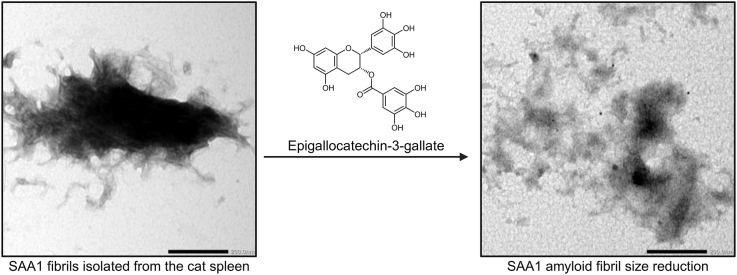

## 1 Introduction

Serum amyloid A1 (SAA1) is a human acute-phase protein that serves as a precursor to amyloid A (AA) fibrils through proteolysis [1,2]. It is primarily produced by the liver in response to proinflammatory cytokines during acute inflammation [3]. SAA1 consists of a 122-amino acid structure, which matures into a 104-amino acid protein after the cleavage of its signal peptide [4]. The SAA1 N-terminus facilitates high-density lipoprotein (HDL) binding, while the SAA1 C-terminus region is responsible for maintaining its structural integrity. In its native state, SAA1 forms a hexamer composed of four-helix bundles and plays a role in lipid metabolism through HDL remodeling, as well as in regulating the immune response [5].

Elevated levels of SAA1 are linked to an increased risk of AA amyloidosis, a condition secondary to chronic inflammation and infection. This disease state is characterized by the accumulation of insoluble AA fibril deposits in the body in a β-pleated sheet conformation. The transformation of amyloid-like fibrils involves a structural change in the N-terminal region of SAA1 from an alpha-helix to a beta-sheet configuration [6]. AA amyloidosis affects various animal species and may be transmissible between them through intravenous, intraperitoneal, or even oral routes (e.g., amyloid-contaminated food) [7]. In waterfowl, these amyloid deposits are commonly found in the liver, spleen, kidneys, pancreas, intestine, thyroid gland, heart, and lungs [8]. In cats, amyloid deposits are commonly found in the spleen, kidneys, and liver [9]. Notably, the liver is the main target of amyloids in Siamese and Oriental cats [10,11], while the kidney is the main site in the Abyssinian cat [12]. These amyloid deposits have been reported to affect the following organs in cattle: kidneys, liver, spleen, gastrointestinal tract, and adrenal glands [9].

Metal complexes and natural polyphenolic compounds have been studied for their potential to inhibit amyloid aggregation and reduce the associated effects. In particular, two η6-arene Ru(II) complexes were investigated for their impact on amyloid aggregation and were found to reduce aggregation while stabilizing peptide structures in a more helical conformation [13]. Similarly, secoiridoid derivatives from olive oil polyphenols, such as hydroxytyrosol, tyrosol, oleuropein, oleocanthal, and oleacein, exhibit a broad spectrum of biological properties, including anticancer, antidiabetic, antioxidant, anti-steatotic, cardioprotective, and neuroprotective activities. More specifically, hydroxytyrosol functions as a potent free radical scavenger and metal chelator to alleviate oxidative stress [14]. Its anticancer effects are associated with the suppression of protein kinase B and nuclear factor-kappa B signaling pathways, resulting in decreased tumor growth and angiogenesis. Additionally, hydroxytyrosol's ability to inhibit toxic protein aggregates suggests its potential use in amyloid-related diseases.

Epigallocatechin-3-gallate (EGCG), a bioactive polyphenolic flavonoid found in green tea, is renowned for its antioxidant properties and is widely recognized as an anti-amyloidogenic agent against aggregation-prone proteins, including amyloid-beta 42 (Aβ42) [[15], [16], [17], [18]], alpha-synuclein (α-syn) [15,16,19], islet amyloid polypeptide (IAPP) [20], transthyretin (TTR) [21,22], huntingtin [23], tau [24], immunoglobulin light chain [25], kappa-casein [26], and microcin E492 [27]. Additionally, EGCG was previously found to inhibit fibril formation of SAA1 in cell culture [28]; however, there is no current information regarding EGCG's ability to disaggregate SAA1 fibrils. For this reason, EGCG is studied here for its application to disaggregate SAA1 fibrils isolated from animals. Several studies have demonstrated EGCG's ability to disaggregate mature fibrils of other aggregation-prone proteins, particularly Aβ42, α-syn, IAPP, TTR, and tau, into smaller, amorphous, non-toxic aggregates, thereby reducing their cellular toxicity [15,20,22,29]. Mechanistically, EGCG binds to unfolded polypeptide chains, preventing β-pleated sheet formation and potentially redirecting aggregation-prone polypeptides to non-toxic pathways [16]. Specifically, EGCG prevents the conversion of α-syn from random coils into β-sheets by binding to isoleucine (Ile), phenylalanine (Phe), and tyrosine (Tyr) residues [30]. Additionally, it disaggregates preformed α-syn amyloid fibrils by interacting with leucine (Leu), histidine (His), phenylalanine (Phe), and tyrosine (Tyr) residues, and protects PC12 cells from α-*syn*-induced cytotoxicity by reducing reactive oxygen species production [30]. Similarly, EGCG can alter the conformational structure of Aβ42, increasing its random coil content and disrupting β-sheet formation with preferential binding to its hydrophobic cavity [31]. Building on EGCG's disaggregation abilities, researchers developed **CNS-11** and **CNS-11g**, compounds designed using the EGCG pharmacophore as a docking site; these compounds feature a central amide backbone with low polarity to allow for blood-brain barrier penetration [32]. Both **CNS-11** and **CNS-11g** were able to disaggregate α-syn, reduce seeding aggregation, lower fibril counts and aggregate number, and shorten fibril length, potentially through destabilization of N-terminal residues within the fibril core.

In recent years, the incidence of AA amyloidosis in humans has declined due to reductions in chronic infections and improvements in the treatment of chronic inflammatory disorders [33]. Despite these advancements, no effective treatment has been developed to specifically target AA fibril deposits. To address this challenge, we aim to investigate the response of SAA1 fibrils to EGCG. For this purpose, we isolated SAA fibrils from the diseased spleen of a cat (*Felis catus*), as well as from the diseased liver tissue of a cow (*Bos taurus*) and a chicken (*Gallus gallus domesticus*). These natural fibrils in diseased tissue exhibit structural differences compared to those formed by synthetic peptides or produced by recombinant protein production in bacteria [34]. To further confirm its impact, we also studied the effects of EGCG on Aβ fibrils isolated from Alzheimer's disease (AD) brains. Identifying agents that can disaggregate SAA1 fibrils could significantly advance the therapeutic management or prevention of inflammatory diseases and neurodegenerative diseases.

## 2 Materials and methods

### 2.1 Chemicals

EGCG was obtained from Sigma Aldrich (St. Louis, MO) with purity levels of ≥95 %.

### 2.2 Sample preparation

Fibrils were isolated from the cat spleen, chicken liver, and cow liver using a water extraction method [35]. The fibrils were dried after purification and stored at room temperature. These fibrils were subsequently solubilized in PBS (pH 7.4) and were further sonicated. The sonication was carried out for approximately 5 min, using a cycle of 30 s on and 1 min off, at a power setting ranging between 30 % and 45 %. The resulting mixture was then used as a working stock solution for subsequent experiments. Protein quantification was performed using the Lowry method in the prepared stock solution. The protein concentrations of the original stock solution for each fibril are as follows: cat spleen – 21.0 ± 2.8 mg/mL; chicken liver – 16.5 ± 1.4 mg/mL; cow liver – 29.7 ± 2.1 mg/mL.

Concerning the SAA1 fibril sample treatments, the fibrils original stock solution from each species were diluted to 1:10 using 10 mM phosphate-buffered saline (PBS) at pH 7.4 prior to sample treatment. The different fibril treatments were prepared by pipetting 8.5 μL from the 1:10 diluted stock of the isolated fibrils in PBS in a total volume of 100 μL with 1 % dimethyl sulfoxide (DMSO, control) and 400 μM EGCG (from a 40 mM stock in 100 % DMSO). The final solutions analyzed by dynamic light scattering and TEM consisted of the total protein concentrations as follows: cat spleen – 178.8 μg/mL; chicken liver – 140.6 μg/mL; cow liver – 252.5 μg/mL.

The A*β* plaques were purified from post-mortem human AD brains obtained from patients diagnosed with advanced Alzheimer's disease (Braak stage V or VI; Tissue Biobank at Case Western Reserve University-Cleveland Clinic). The A*β* plaques were isolated using ultracentrifugation with a sucrose gradient and cell sorting [25,26]. Purified A*β* plaques were examined with polarized and fluorescence microscopy using Congo Red and thioflavin-S staining. Validation was done with electron microscopy. Each aliquot of A*β* plaques was solubilized with 60 μL of 10 mM PBS buffer (pH 7.4). The extraction consisted of 0.51 ± 0.11 mg/mL of proteins, as measured by the Lowry method. A volume of 20 μL of extraction was incubated with 1.5 % DMSO (control) or 50 μM of EGCG for 5 days at 37 °C.

### 2.3 Dynamic light scattering

Dynamic light scattering was performed using the Malvern Zetasizer to analyze the particle size of each sample. Fibrils from the cat spleen, chicken liver, and cow liver were incubated in 10 mM PBS (pH 7.4, 37 °C) and centrifuged for 15 min at 150,000 rpm with either 1 % DMSO or 400 μM EGCG in microcentrifuge tubes. 50 μL from each supernatant sample was then transferred to a low-volume quartz cuvette (QS high precision cell, 105-251-85-40, Hellma Analytics) and measured at 37 °C using the fluorescence filter at time zero, and after seven days for the cat spleen, six days for the chicken liver, and five days for the cow liver samples. Each measurement was performed in triplicate. The results represent the soluble particles and were analyzed in comparison to the non-centrifuged samples to assess significant changes.

### 2.4 Transmission electron microscopy

Transmission electron microscopy of the soluble and insoluble fibrils was utilized to examine amyloid fibrils extracted from cat spleen, chicken liver, and cow liver. Fibrils prepared in PBS with either 1 % DMSO or 400 μM EGCG were incubated at 37 °C for five days. After incubation, 10 μL of the fibril suspension was vortexed and placed onto a 400-mesh Formvar-carbon-coated copper grid (Electron Microscopy Sciences, Hatfield, PA). The grids were incubated for 1 min, rinsed with distilled water, and air-dried. They were then further incubated for 1 min in fresh 1 % uranyl acetate before being air-dried again. Fibril visualization was performed using TEM (JEOL 1400 Flash, Japan) at 100 kV with magnifications of 2500x and 40k.

Quantitative measurement of plaque density was conducted to support the observed changes in size. The areas of eight different plaques for each sample were analyzed using ImageJ software. These measurements were then plotted and analyzed using GraphPad Prism 9, providing statistical validation of the plaque size reduction after treatment.

Transmission electron microscopy was also used to analyze the soluble portion of the cow and chicken liver samples from the DLS analysis. Fibrils from the cow and chicken liver were incubated in 10 mM PBS (pH 7.4, 37 °C) at a concentration of 10 μM and then centrifuged for 15 min at 150,000 rpm with either 1 % DMSO or 400 μM of EGCG. Grids were prepared from the supernatant containing soluble or semi-soluble particles. Fibril visualization was performed using the same JOEL TEM.

Further, transmission electron microscopy was used to analyze the effects of EGCG on amyloid plaques from AD brains. The amyloid plaques were incubated in 10 mM PBS (pH 7.4, 37 °C) with either 0.25 % DMSO or 50 μM of EGCG for five days at 37 °C. Grids were prepared and evaluated in the same manner as described above from the amyloid plaques.

## 3 Results

**Fig. 1:**
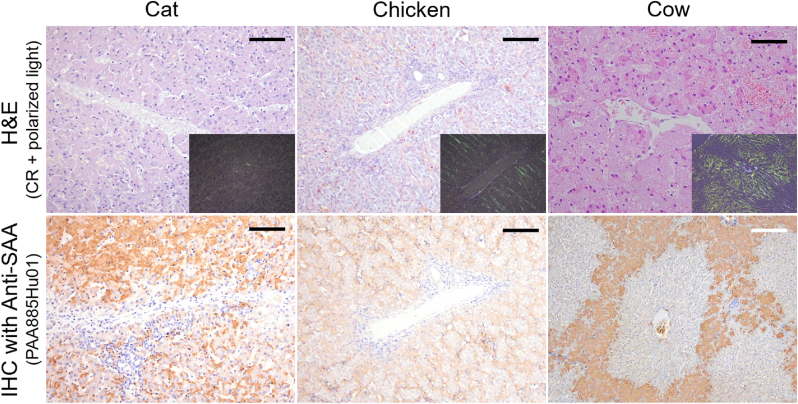
Photomicrographs of the cat spleen, chicken liver, and cow liver histological sections stained with hematoxylin & eosin (H&E), Congo red (CR, polarized light) and SAA immunohistochemistry confirmed the presence and nature of amyloid deposits. Pictures were acquired at a magnification of 20X. Black scale bar: 100 μm; White scale bar: 500 μm.

Prior to experiments, the nature of the isolated fibrils from the organs affected by amyloid deposits and obtained post-mortem from different animal species was confirmed by histopathological examination, immunohistochemical analysis, and Western blot. Amyloid deposits were present in the hepatic sinusoids of the cat, chicken, and cow (Fig. 1, hematoxylin & eosin-stained sections (H&E)). The histological sections were stained with Congo red (CR) and pictures were acquired under polarized light to demonstrate the characteristic yellow green birefringence. Each CR-stained histological section from the cat, chicken, and cow liver demonstrated the birefringence (Fig. 1, inserted smaller figures). Additionally, the immunohistochemistry of the histological sections using anti-SAA showed positive signal for each species (Fig. 1). Western blot analyses were performed with the isolated fibrils. The results are shown in Fig. S1 (supplemental data section). High molecular weight bands were immunoreactive with the SAA1 monoclonal antibody for the samples isolated from the cat spleen, chicken liver, and cow liver. These bands represent the multimerization of the SAA1 protein.

### 3.1 Dynamic light scattering analysis of particles

Dynamic light scattering (DLS) was used to assess the particle size of the cat spleen, chicken liver, and cow liver SAA1 fibrils in solution. Light scattering on particles using laser and correlogram allows for the determination of the size of particles in solution. The samples obtained from different animal species were first solubilized in 10 mM PBS (pH 7.4) at a final concentration between 1 and 2 mg/mL (original stock concentrations: cat spleen – 21.0 mg/mL; cow liver – 29.7 mg/mL; chicken liver – 16.5 mg/mL). After a 1:100 dilution in PBS, the samples containing aggregates were treated with 1 % DMSO or 400 μM EGCG and incubated at 37 °C for five to seven days. The samples were centrifugated and the supernatant was analyzed. This portion corresponds to the soluble and partially soluble fraction of the isolated aggregates. The effect of EGCG at a lower concentration (i.e. 200 μM) was assessed but provided the least prominent changes as demonstrated in Fig. S2 and Table S1 (supplemental data).

**Fig. 2:**
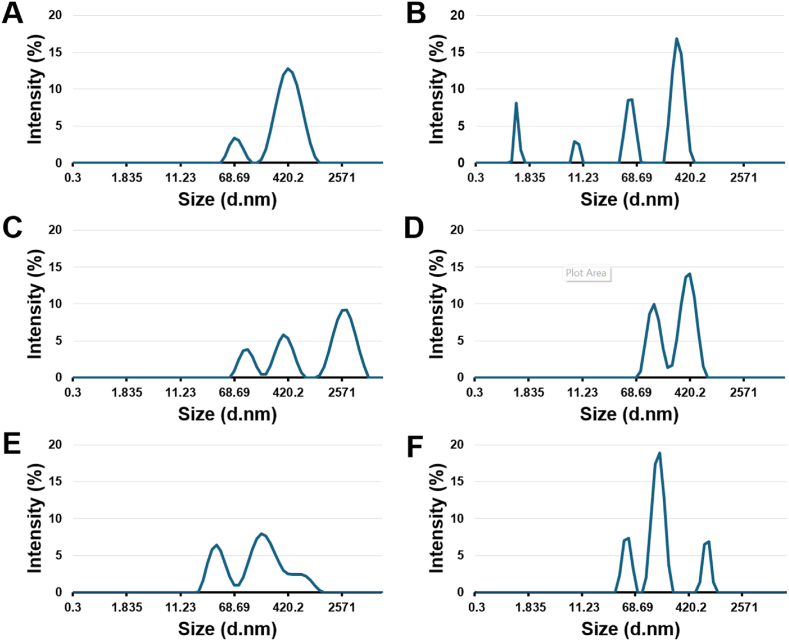
Dynamic light scattering (DLS) analysis of SAA1 fibril samples extracted from post-mortem specimens: cat spleen, chicken liver, and cow liver. The samples were incubated at 37 °C for seven (cat), six (chicken), and five (cow) days in either 1 % DMSO or 400 μM EGCG, prepared in 10 mM PBS (pH 7.4). The samples were centrifuged at 150,000 rpm for 15 min, and the supernatant was analyzed with the Zetasizer Pro (Malvern). DLS graphs of the intensity (percent) in function of the size of particles (diameter, nm) resulting from the supernatant of the **A)** DMSO-treated cat spleen SAA1 fibrils; **B)** EGCG-treated cat spleen SAA1 fibrils; **C)** DMSO-treated chicken liver SAA1 fibrils; **D)** EGCG-treated chicken liver SAA1 fibrils; **E)** DMSO-treated cow liver SAA1 fibrils; **F)** EGCG-treated cow liver SAA1 fibrils.

The DLS analysis of chicken liver SAA1 fibrils revealed a leftward shift following 400 μM EGCG treatment (Fig. 2). The EGCG treated cat and cow SAA1 fibrils resulted in less broad and intense peak in the region representing particles of high nanometer (nm) in diameter. The cat spleen fibrils shifted from peaks at 70 nm and 420 nm with the control 1 % DMSO to peaks at 1 nm, 8 nm, 50 nm, and 270 nm after the treatment with 400 μM EGCG. The chicken liver fibrils, which initially showed peaks at 110 nm, 360 nm, and 3000 nm with the control (1 % DMSO, vehicle), shifted to 130 nm and 420 nm upon treatment with 400 μM EGCG, accompanied by the disappearance of the peak at 3000 nm which is associated with large aggregates. Cow liver fibrils exhibited minimal change, from peaks of 40 nm, 170 nm, and 660 nm with the 1 % DMSO (control) to peaks of 50 nm, 150 nm, and 770 nm after the incubation with 400 μM EGCG. The DLS data also indicated a reduction in the polydispersity index after the treatment with EGCG (from 0.4 (DMSO control) to 0.3 (EGCG) for the cat spleen aggregates, 0.9 (DMSO control) to 0.4 (EGCG) for the chicken liver aggregates, and 0.5 (DMSO control) to 0.4 (EGCG) for cow liver aggregates), suggesting that the samples became more uniform and stable after the EGCG treatments (Table 1). Notably, the Z-average for chicken liver decreased significantly from 430 nm (DMSO control) to 230 nm (EGCG), while the cat spleen showed a smaller reduction, from 270 nm (DMSO control) to 240 nm (EGCG), both indicating a reduction in particle size of protein aggregates after the incubation with 400 μM EGCG. In contrast, cow liver fibrils treated with EGCG showed an increased Z-average compared to those treated with the control (1 % DMSO); however, the peaks are more defined and sharper, indicating the formation of more homogenous protein size populations in solution upon treatment with EGCG.

**Table 1:** Results from the dynamic light scattering (DLS) analysis of SAA1 fibril samples isolated from the cat spleen, chicken liver, and cow liver. The samples were incubated at 37 °C for seven days (cat), six days (chicken), five days (cow) in either 1 % DMSO or 400 μM EGCG. The samples were centrifuged at 150,000 rpm for 15 min, and the supernatant was analyzed with the Zetasizer Pro (Malvern).

	Z-Average	Polydispersity Index (PI)	Peak 1 Mean by Intensity Ordered by Area (nm)	Peak 1 Area by Intensity Ordered by Area (%)	Peak 2 Mean by Intensity Ordered by Area (nm)	Peak 2 Area by Intensity Ordered by Area (%)
Cat Spleen DMSO	270	0.4	462	88	74	12
Cat Spleen EGCG	242	0.3	281	59	56	25
Chicken Liver DMSO	428	0.9	2930	56	380	29
Chicken Liver EGCG	225	0.4	400	63	128	37
Cow Liver DMSO	96	0.5	216	57	41	31
Cow Liver EGCG	306	0.4	141	64	48	20

### 3.2 Transmission electron microscopy analysis of fibrils

To confirm the effects of EGCG treatment in comparison to the control on fibrils, transmission electron microscopy (TEM) was used to directly observe changes in the fibrillar structure of aggregates. Prior to the TEM analysis, the SAA1 fibrils isolated from animal species were incubated in 10 mM PBS (pH 7.4) with either 1 % DMSO or 400 μM EGCG at 37 °C for five days. The concentration of fibril used was identical to the DLS experiments. After incubation, the fibrils were mounted on Formvar-carbon-coated copper grids for imaging.

**Fig. 3:**
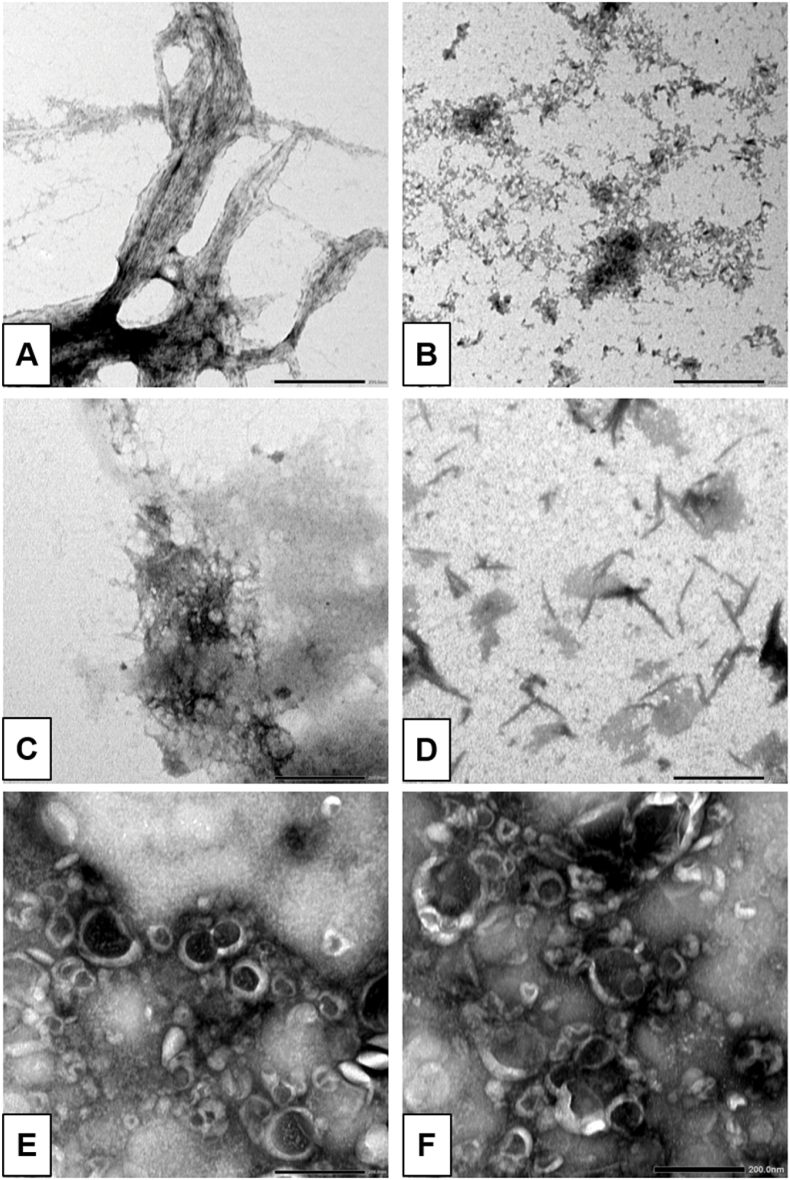
Transmission electron microscopy (TEM) images of chicken and cow liver SAA1 fibrils after incubation with 1 % DMSO or 400 μM EGCG in 10 mM PBS (pH 7.4) at 37 °C for 5 days, using a magnification of 40k. The samples were centrifuged for 15 min at 150,000 rpm, and the supernatant was analyzed. High resolution images (40k magnification) of the **A)** DMSO-treated chicken liver SAA1 fibrils; **B)** EGCG-treated chicken liver SAA1 fibrils; **C)** DMSO-treated cow liver SAA1 fibrils; **D)** DMSO-treated cow liver SAA1 fibrils; **E)** EGCG-treated cow liver SAA1 fibrils; **F)** EGCG-treated cow liver SAA1 fibrils. Scale bar: 200 nm.

The TEM analysis of 400 μM EGCG alone (i.e. without fibrils) was performed as a quality control. Two samples were prepared in 10 mM PBS (pH 7.4) and incubated for 7 days at 37 °C. No particular structures were found on the copper grid. Example of images of EGCG alone are presented in Fig. S3 (supplemental data section). TEM images of SAA1 fibrils from the supernatant portion of chicken and cow liver samples, prepared in the same manner as for DLS and centrifuged, revealed notable ultrastructural changes. The supernatant of the 1 % DMSO-treated chicken and cow fibril samples displayed fibrillar structures (Fig. 3A–C-D). After treatment with 400 μM EGCG, the samples predominantly presented less defined structures for the chicken samples (Fig. 3B). For the EGCG-treated cow liver samples, the fibrils transitioned into round structures of approximately 100 nm in size (Fig. 3E and F), consistent with the DLS measurements (Fig. 2F).

**Fig. 4:**
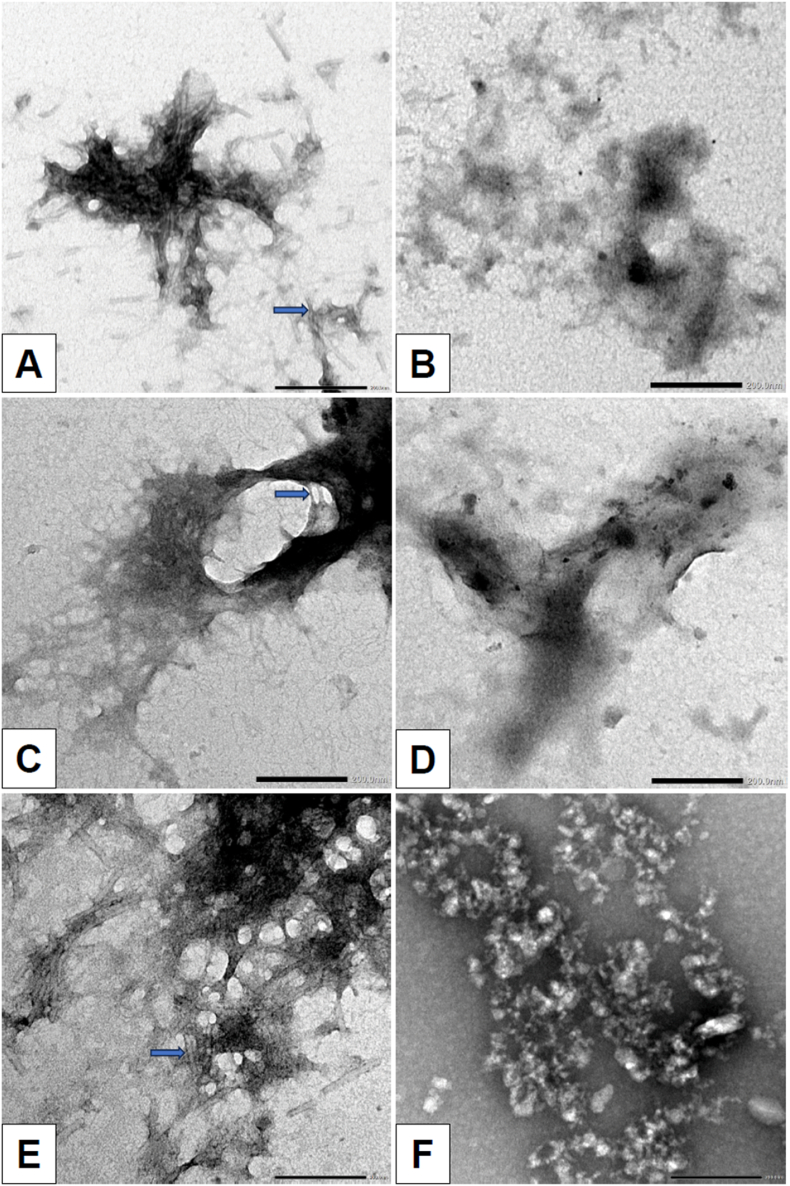
Transmission electron microscopy (TEM) images of SAA1 fibrils of the cat spleen, chicken liver, and cow liver after incubation with 1 % DMSO or 400 μM EGCG in 10 mM PBS (pH 7.4) at 37 °C for 5 days, using a magnification of 40k. Samples were not centrifuged. Total protein concentration for samples analyzed are as follows: cat spleen – 178.8 μg/mL; cow liver – 252.5 μg/mL; chicken liver – 140.6 μg/mL. **A)** TEM image of the DMSO-treated cat spleen SAA1 fibrils. **B)** TEM image of the EGCG-treated cat spleen SAA1 fibrils. **C)** TEM image of the DMSO-treated chicken liver SAA1 fibrils. **D)** TEM image of the EGCG-treated chicken liver SAA1 fibrils. **E)** TEM image of the DMSO-treated cow liver SAA1 fibrils. **F)** TEM image of the EGCG-treated cow liver SAA1 fibrils. The arrows indicate examples of amyloid fibrils. Scale bar: 200 nm.

TEM images of the total fraction of SAA1 fibrils (i.e. non-centrifugated) from the cat spleen, chicken liver, and cow liver treated with 1 % DMSO showed the compact accumulation of fibrils (Fig. 4A–C). Upon treatment with 400 μM EGCG, the cat spleen and chicken liver exhibited less dense accumulation of fibrils (Fig. 4D and E). The EGCG-treated cow liver SAA1 fibrils resulted in less defined structures that are not characteristic of fibrillar structures.

**Fig. 5:**
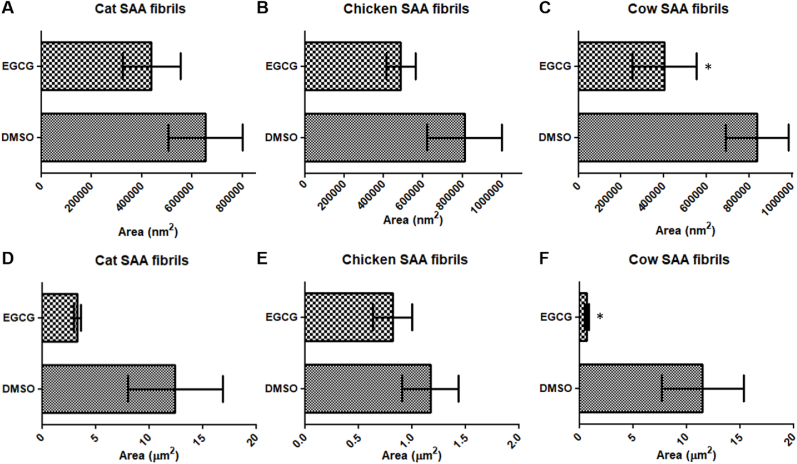
Measurement of SAA1 fibrils of the cat spleen, chicken liver, and cow liver in 1 % DMSO or 400 μM EGCG in 10 mM PBS (pH 7.4) after 5 days incubation at 37 °C. The samples were not centrifuged. Total protein concentration for samples analyzed are as follows: cat spleen – 178.8 μg/mL; chicken liver – 140.6 μg/mL; cow liver – 252.5 μg/mL. The surface areas of the cat spleen, chicken liver, and cow liver aggregates were analyzed using ImageJ and TEM images at a magnification of 20k (A–C). In addition, the surface areas of the aggregates for the cat, chicken, and cow samples were analyzed using TEM images at 2500 magnification (D–F). The histograms allow for comparison of the surface areas as measured by ImageJ of the SAA1 aggregates resulting from DMSO- and EGCG-treated **A)** cat spleen SAA1 fibrils (high magnification); **B)** chicken liver SAA1 fibrils (high magnification); **C)** cow liver SAA1 fibrils (high magnification); **D)** cat spleen SAA1 fibrils (high magnification); **E)** chicken liver SAA1 fibrils (high magnification); **F)** cow liver SAA1 fibrils (low magnification). ∗p < 0.05 by unpaired one-tailed *t*-test.

The surface areas of the various accumulation of fibrils were assessed using ImageJ. Images resulting from the TEM analysis on the non-centrifugated treated SAA1 fibril samples were analyzed. Analysis of the TEM images (at a magnification of 20k) of SAA1 aggregates from the cat spleen, chicken liver, and cow liver, treated with EGCG in comparison with the control (1 % DMSO), revealed a reduction of the area of compact accumulation of fibrils (Fig. 5). The results showed a trend for the cat and chicken, but were statistically significant for the cow fibrils exposed to EGCG (Fig. 5C–F). Specifically for the TEM images acquired at low magnification (i.e. 2500) for the cow liver samples, the area of fibril accumulation decreased from 4.9 ± 1.9 μm^2^ (control DMSO) to 0.4 ± 0.1 μm^2^ (EGCG treatment) (Fig. 5F). The TEM images in Fig. 4 show the reduction of the fibril accumulation in density and/or size with EGCG-treated samples in comparison to the control (1 % DMSO-treated samples). Examples of TEM images and area measurements of accumulation of fibrils with ImageJ are presented in the supplemental data section (Fig. S4–S6).

**Fig. 6:**
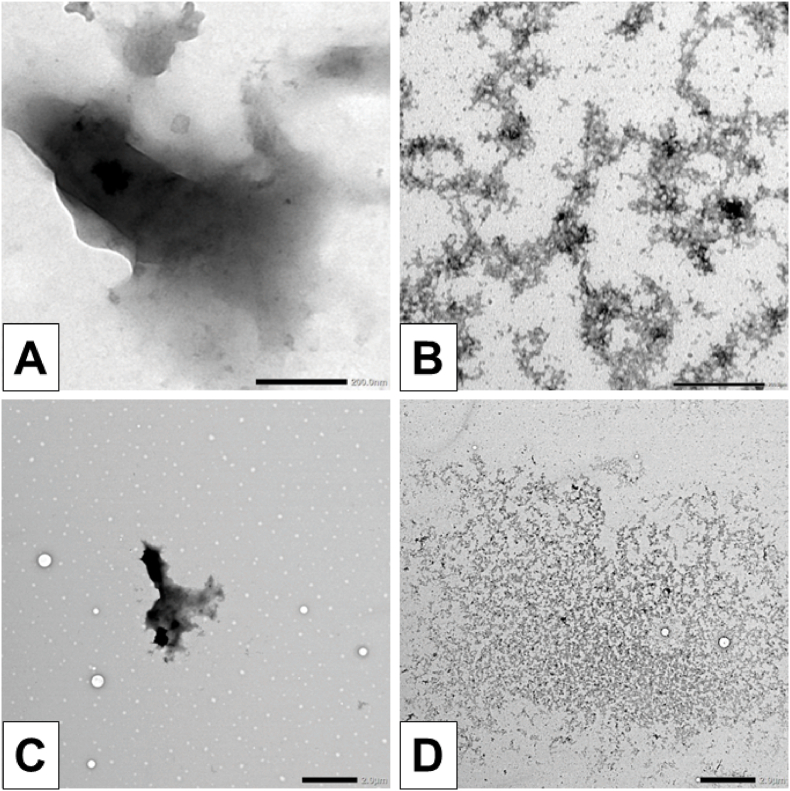
Transmission electron microscopy (TEM) images of amyloid plaques isolated from Alzheimer's disease (AD) brains after incubation in 0.25 % DMSO or 50 μM EGCG in 10 mM PBS (pH 7.4) at 37 °C for five days. The samples were not centrifuged prior to grid preparation. The extraction consisted of 0.5 ± 0.1 mg/mL of proteins. **A)** TEM image of DMSO-treated Aβ dense aggregates isolated from AD brains at high magnification (40k). Scale bar 200 nm. **B)** TEM image of EGCG-treated amyloid plaques isolated from the AD brain at high magnification (40k). Scale bar 200 nm. **C)** TEM image of DMSO-treated Aβ dense aggregates isolated from AD brains at low magnification (2500). Scale bar 2 μm. **D)** TEM image of EGCG-treated Aβ dense aggregates isolated from the AD brain at low magnification (2500). Scale bar: 2 μm.

The impact of EGCG on disaggregating aggregation-prone proteins was further confirmed through its effect on Aβ fibrils isolated from the AD brain. Extraction consisted of 0.51 ± 0.11 mg/mL of proteins. Amyloid plaques isolated from the AD brain were treated with 0.25 % DMSO (control) and 50 μM EGCG. Samples were incubated for five days at 37 °C. TEM images of Aβ fibrils treated with 0.25 % DMSO resulted in dense aggregates (Fig. 6) and fibrils (Fig. S7). However, following EGCG treatment, these dense accumulation of aggregates and fibrils transitioned into not-well defined structures, possibly due to enhanced dense aggregate breakdown. Additional TEM images which demonstrate the presence of fibrillar structures in the 0.25 % DMSO-treated Aβ sample are presented in Fig. S7 (supplementary document).

## 4 Discussion

The combined data from DLS and TEM analyses indicate that EGCG is effective in reducing amyloid-like fibril size and disaggregating SAA1 fibrils. DLS analysis showed a reduction in size for cat spleen and chicken liver fibrils, and sharper, less broad peaks for cow liver fibrils. TEM imaging further confirmed that EGCG breaks down SAA1 fibrils from the cat spleen, chicken liver, and cow liver, facilitating the transition of these fibrils into soluble, smaller and less defined structures. Additionally, TEM analysis of amyloid plaques from AD brains suggests that EGCG may be effective and versatile in disaggregating other aggregation-prone proteins such as Aβ, offering potential therapeutic benefits for neurodegenerative diseases.

These results are consistent with previous studies demonstrating that EGCG can induce the conversion of Aβ and α-syn fibrils into smaller, unstructured, nontoxic aggregates [15]. Both Aβ and α-syn are implicated in the pathogenesis of neurodegenerative diseases through their aggregation into neurotoxic structures. Building on these findings, future research could explore the ability of EGCG and other bioactive gallate-containing compounds to potentially disaggregate other proteins prone to abnormal pathogenic aggregation, such as tau and IAPP. Targeting these proteins may reveal that EGCG or similar compounds could be used therapeutically to remodel amyloids, offering treatment options for conditions like AA amyloidosis, AD, type 2 diabetes, and others. EGCG extracts and formulations have been tested in more than 60 clinical trials. Additional clinical trials are testing safety and tolerance of EGCG extracts, and for potential applications in AD, Parkinson's disease, multiple sclerosis, and other non-neurological conditions.

When determining how EGCG may be used pharmacologically, it is important to discuss a few key findings regarding its mechanism when interacting with proteins like tau and α-syn, which are involved in neurodegenerative diseases such as Alzheimer's and Parkinson's disease. The wedge-charge-destabilization hypothesis suggests that EGCG fits into a wedge-shaped cleft between two protofilaments of tau and disrupts the formation of fibrils [36]. It does this by increasing charge repulsion between the fibril layers, preventing them from sticking together, with an affinity of 1.6 μM [36]. Moreover, EGCG may inhibit the aggregation of α-syn (involved in Parkinson's physiopathology and other neurodegenerative diseases) by interacting through unstable intermolecular hydrophobic interactions [37]. This suggests that for EGCG to effectively prevent aggregation, it may need to be formulated with hydrophobic structures to minimize water interaction, to preserve its anti-aggregation effects. Furthermore, due to its vicinal trihydroxy structure, EGCG can reduce oxidative damage by chelating metal ions such as copper and iron, forming stable complexes [38]. Previous research has demonstrated that copper chelators can destabilize high-order amyloid structures, breaking them down into smaller fibrillary aggregates [39]. This metal-chelating ability of EGCG may contribute to its ability to disaggregate amyloid fibrils, offering a potential therapeutic approach for neurodegenerative and inflammatory diseases associated with amyloid aggregation.

One major concern regarding the use of EGCG as a disaggregating agent for formed SAA1 fibrils is that the disassembly of fibrils into intermediate oligomeric species may be more cytotoxic and damaging than the mature amyloid fibrils themselves [40]. However, EGCG has been found to disaggregate pre-formed oligomers, promote the conversion of toxic oligomers into less toxic fibrils, and inhibit the aggregation of proteins into toxic oligomers [41,42]. To specifically test the effects of EGCG disaggregation on SAA1 oligomer formation in future studies, a Western blot using the anti-oligomer antibody can be employed to evaluate the disaggregation effect of EGCG on these isolated fibrils. However, the antibody will need to be validated with SAA1 oligomers. Additionally, oligomer formation can be assessed using size exclusion chromatography coupled with mass spectrometry.

There are also concerns about the efficacy of EGCG in amyloid-related disorders. A major study that raised this concern was the PROMESA clinical trial, in which patients with multiple system atrophy (MSA) received 400 mg oral EGCG daily for four weeks, then 800 mg for four weeks, and finally 1200 mg daily for forty weeks [43]. The study found no change in disease progression and reported hepatotoxicity at the 1200 mg dosage compared to placebo. However, an observational study involving patients with cardiac TTR amyloidosis assessed the effects of daily consumption of approximately 500–700 mg of EGCG over 12 months [44]. Participants achieved this dosage by either drinking 1.5–2 L of green tea daily or taking caffeine-free green tea extract capsules. The study found no progression of left ventricular wall thickness or left ventricular myocardial mass and also reported reductions in total cholesterol and LDL cholesterol. Though these two clinical trials deliver conflicting results, it is important to note that each targets a different disease state in which the aggregating protein deposits in different organs. Therefore, it is necessary to continue investigating the relevance of EGCG in amyloid-related disorders.

There are a few limitations to this study. Firstly, the area measurements for the cat spleen and chicken liver aggregates may have detected regions with less dense population of fibrils, leading to an inaccurate representation of the disaggregation effects of EGCG on these fibrils. There were also some instances of impossibility to capture all fibril areas with the ImageJ program. Additionally, the study focused exclusively on the effects of EGCG on proteins isolated from post-mortem tissues from the cat spleen, chicken liver, and cow liver. To gain a more comprehensive understanding of EGCG's potential therapeutic effects, it would be beneficial to evaluate its impact on animal models and post-mortem human tissues.

## 5 Conclusion

The combined findings from the DLS analysis and TEM imaging demonstrate that EGCG is effective in altering the size and structure of amyloid fibrils in the diseased animal tissue from the cat spleen, cow liver, and chicken liver. DLS analysis revealed that EGCG treatment reduced the size of aggregates for chicken liver and cat spleen SAA1 fibril samples and improved uniformity for cow liver SAA1 fibril samples, as indicated by sharper, less broad peaks. TEM imaging confirmed that EGCG leads to the breakdown of SAA1 fibrils, converting them into less dense fibrillar structures. Furthermore, in relation to neurodegeneration, EGCG effectively disaggregated Aβ fibrils isolated from AD brains. These findings show EGCG's potential as a therapeutic agent in targeting amyloid fibrils and promoting disaggregation. Future research should focus on its efficacy against other aggregation-prone proteins associated with neurodegenerative diseases and chronic conditions, as well on conducting long-term studies to determine the fate of amyloid fibril fragments after disaggregation.

## Funding

This study was funded by the ASIP Summer Research Opportunity Program in Pathology (SROPP) and the 2024 Breakthrough Seed Award – Purdue Honors College.

## CRediT authorship contribution statement

**Natalie G. Horgan:** Conceptualization, Data curation, Formal analysis, Investigation, Writing – original draft, Writing – review & editing. **Anabela Djurovic-topalovic:** Data curation, Formal analysis, Investigation. **Taiwo A. Ademoye:** Data curation, Formal analysis, Investigation. **Hannah I. Reyes-Charles:** Data curation, Formal analysis, Investigation, Writing – review & editing. **Natsumi Kobayashi:** Data curation, Formal analysis, Writing – review & editing. **Germán Plascencia-Villa:** Data curation, Formal analysis, Writing – review & editing. **George Perry:** Data curation, Formal analysis, Writing – review & editing. **Tomoaki Murakami:** Data curation, Formal analysis, Writing – review & editing. **Jessica S. Fortin:** Conceptualization, Data curation, Formal analysis, Funding acquisition, Investigation, Project administration, Supervision, Validation, Writing – review & editing.

## Declaration of competing interest

The authors declare that they have no known competing financial interests or personal relationships that could have appeared to influence the work reported in this paper.

## Data Availability

Data will be made available on request.
