# Supplementary material for: Epigallocatechin-3-Gallate: A potential amyloid Fibril Disaggregator of Serum amyloid A1
Source: Biochem Biophys Rep. 2025 Nov 16;44:102365. doi: 10.1016/j.bbrep.2025.102365 (PMC12664600; doi:10.1016/j.bbrep.2025.102365)
Supplement: Multimedia component 1 [file mmc1.docx]

**Epigallocatechin-3-Gallate: A Potential Amyloid Fibril Disaggregator of Serum Amyloid A1**

Natalie G. Horgan^a^, Anabela Djurovic-topalovic^a^, Taiwo A. Ademoye^a^, Hannah I. Reyes-Charles^a^, Natsumi Kobayashi^b^, Germán Plascencia-Villa^c^, George Perry^c^, Tomoaki Murakami^b^, and Jessica S. Fortin^a,^*

^a^Basic Medical Sciences, College of Veterinary Medicine, Purdue University, 625 Harrison Street, West Lafayette IN 47907, USA

^b^Laboratory of Veterinary Toxicology, Tokyo University of Agriculture and Technology, 3-5-8 Saiwai-cho, Fuchu, Tokyo, Japan

^c^Deptartment of Neuroscience, Developmental and Regenerative Biology, The University of Texas at San Antonio, San Antonio, TX, 78249, USA

***Contact** Jessica S. Fortin fortinj@purdue.edu Department of Basic Medical Sciences, College of Veterinary Medicine, Purdue University, 610 Purdue Mall, West Lafayette, IN 47907

**INDEX**

Western blotting of SAA1 fibril samples………………………………………………..p. S1-S2

Analysis of SAA1 fibrils by dynamic light scattering…….………….……….…….…..p. S2-S4

Analysis of EGCG by transmission electron microscopy……………………………….p. S4

Example of SAA1 fibril measurements with ImageJ……………………………………p. S4-S7

Analysis of amyloid plaques by transmission electron microscopy…………….……….p. S8

**Western blotting of SAA1 fibril samples.** To evaluate the nature of the fibrils (i.e., if they are from the SAA1 protein), western blotting was performed on fibril preparations isolated from the cat spleen, chicken liver, and cow liver, all of which had amyloidosis. The samples loaded consisted of a small volume of 5 µL from each protein sample and 8.33 µL of 3X Laemmli buffer (with 15% β-mercaptoethanol). The entire volume was loaded onto a 16% SDS-Page gel. After electrophoresis, the protein was transferred on a nitrocellulose membrane for western blot analyses. The primary antibody, SAA1 monoclonal mouse IgG1 clone #CL9175 (NPB3-21169, Bio-Techne), was diluted 1:100 in 5% non-fat milk. The nitrocellulose membrane was incubated with the diluted antibody overnight at 4 °C. After washing the nitrocellulose membrane with 0.1% TBST, the membrane was incubated with a secondary antibody (anti-mouse coupled with horseradish peroxidase, HRP) diluted 1:2500 in 5% non-fat milk for one hour at room temperature. Pierce ECL Western Blotting Substrate (cat # 33209, Thermo scientific) was used to detect the HRP signal. **Figure S1** shows the presence of multimeric and high molecular weight species in the fibril samples isolated from the cat spleen, chicken liver, and cow liver, which exhibit immunoreactivity with the mouse SAA1 antibody.


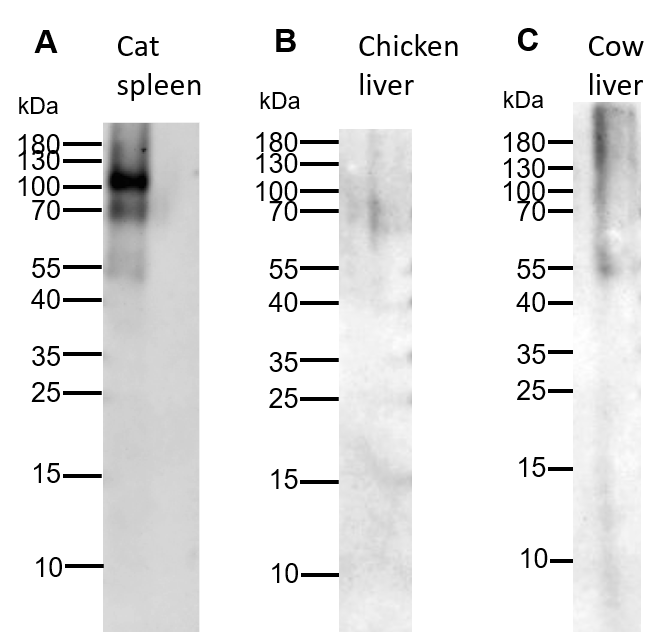


**Figure S1.** SAA1 fibrils isolated from cat spleen, chicken liver, and cow liver as validated by Western blot. The fibril sample original stock solutions were prepared with Laemmli buffer and loaded onto a 16% SDS-PAGE gel. Proteins were transferred on a nitrocellulose membrane and the SAA1 monoclonal antibody (NPB3-21169, Bio-Techne) was utilized during the western blot procedure. High molecular weight species representing multimeric SAA1 protein were detected from each sample isolated from the cat spleen, chicken liver, and cow liver.

**Analysis of SAA1 fibrils by dynamic light scattering (DLS).** To evaluate the efficacy of EGCG at 200 µM and 400 µM, DLS analysis was performed. Fibril stock samples were diluted at 1:100 in 10 mM PBS supplemented with 1% DMSO, 200 μM EGCG, or 400 μM EGCG. After 46 hours of incubation at 37 °C, the samples were centrifuged at 150,000 rpm for 15 minutes. The supernatant was read using the zetasizer. The intensity (percent) and size (diameter in nm) of the particles from each sample are presented in **Figure S2** as well as additional parameters in **Table S1**. The 400 µM EGCG treatment induced a more pronounced leftward shift than the 200 µM EGCG treatment. Concerning the control 1% DMSO (vehicle), the chicken liver fibrils exhibited peaks at 20 nm, 170 nm, and 770 nm, which shifted to 40 nm, 150 nm, and 570 nm after the treatment with 200 µM EGCG and to 1 nm and 20 nm after the treatment with the higher concentration of EGCG, i.e. 400 µM. The cow liver fibrils control (1% DMSO) initially resulted in peaks at 40 nm and 200 nm. These peaks shifted to 80 nm, 230 nm, and 1200 nm after the treatment with 200 µM EGCG and to 30 nm, 80 nm, and 360 nm after the incubation with 400 µM EGCG. The cat spleen fibrils incubated with 1% DMSO (control, vehicle) resulted in initial peaks at 110 nm and 420 nm in DMSO. Those peaks shifted to 150 nm, 900 nm, and 5500 nm after exposure with 200 µM EGCG and to 2 nm, 40 nm, 170 nm, and 900 nm after the treatment with 400 µM EGCG.

**
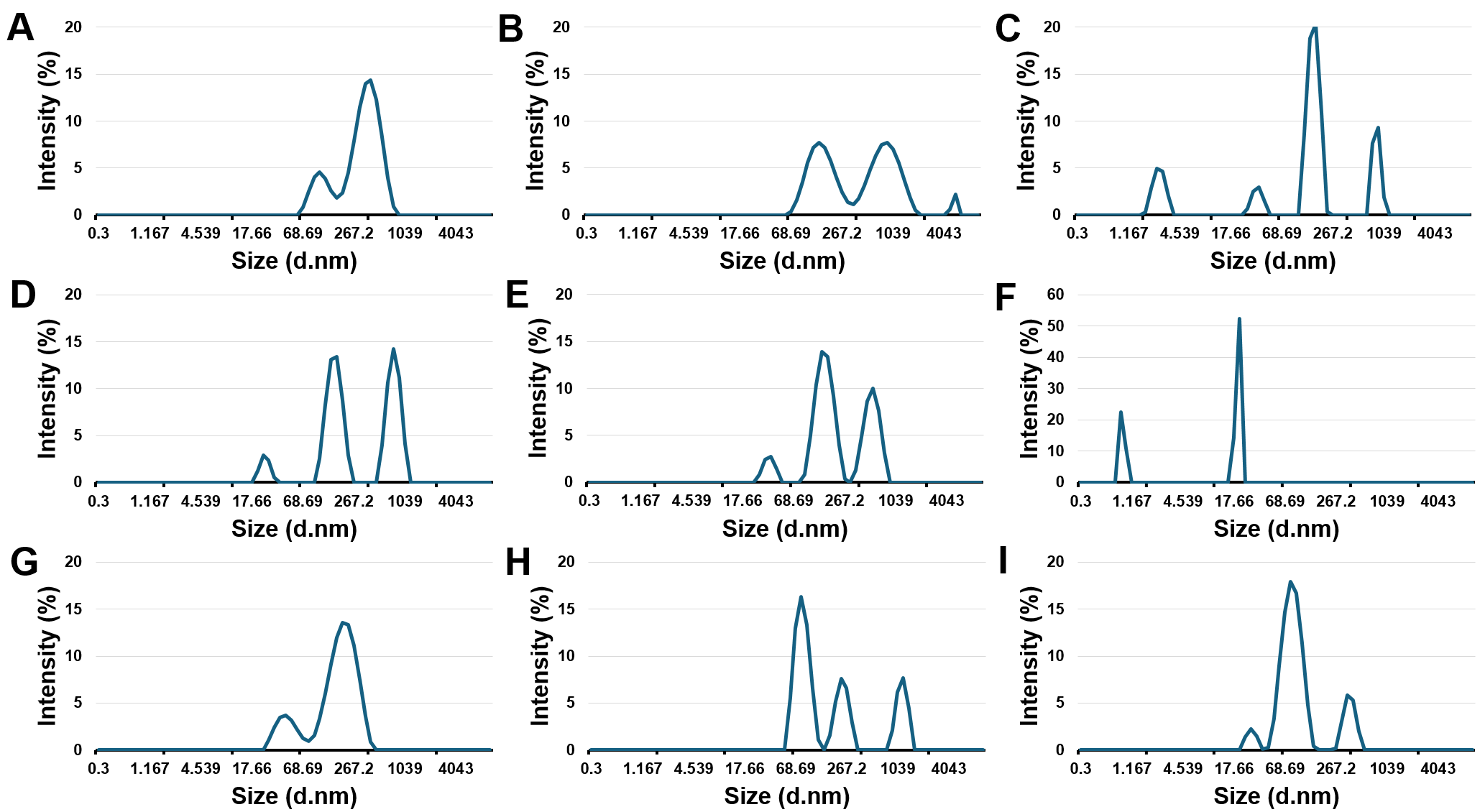
**

**Figure S2.** Dynamic light scattering (DLS) analysis of SAA1 fibril samples derived from the cat spleen, chicken liver, and cow liver. The samples diluted (1:100) in 10 mM PBS (pH 7.4) were incubated at 37 °C for 46 hours with 1% DMSO, 200 μM EGCG, or 400 μM EGCG. The samples were centrifuged at 150,000 rpm for 15 minutes, and the supernatant was analyzed. Each graph shows the intensity of the peaks (percentage) in function of the size of the particles (diameter in nm). DLS results of the: **A)** cat spleen SAA1 fibrils incubated with 1% DMSO; **B)** cat spleen SAA1 fibrils incubated with 200 μM EGCG; **C)** cat spleen SAA1 fibrils incubated with 400 μM EGCG; **D)** chicken liver SAA1 fibrils incubated with 1% DMSO; **E)** chicken liver SAA1 fibrils incubated with 200 μM EGCG; **F)** chicken liver SAA1 fibrils incubated with 400 μM EGCG; **G)** cow liver SAA1 fibrils incubated with 1% DMSO; **H)** cow liver SAA1 fibrils incubated with 200 μM EGCG; **I)** cow liver SAA1 fibrils incubated with 400 μM EGCG.

|  | Z-Average | Polydispersity Index (PI) | Peak 1 Mean by Intensity Ordered by Area (nm) | Peak 1 Area by Intensity Ordered by Area (%) | Peak 2 Mean by Intensity Ordered by Area (nm) | Peak 2 Area by Intensity Ordered by Area (%) |
| --- | --- | --- | --- | --- | --- | --- |
| Cat Spleen 1% DMSO | 244 | 0.5 | 399 | 80 | 114 | 20 |
| Cat Spleen 200 μM EGCG | 282 | 0.6 | 891 | 50 | 164 | 47 |
| Cat Spleen 400 μM EGCG | 601 | 0.5 | 161 | 59 | 857 | 19 |
| Chicken Liver 1% DMSO | 401 | 0.4 | 162 | 49 | 781 | 44 |
| Chicken Liver 200 μM EGCG | 224 | 0.4 | 159 | 57 | 559 | 36 |
| Chicken Liver 400 μM EGCG | 4260 | 1 | 20 | 67 | 0.9 | 34 |
| Cow Liver 1% DMSO | 143 | 0.4 | 210 | 82 | 47 | 18 |
| Cow Liver 200 μM EGCG | 249 | 0.4 | 83 | 56 | 239 | 24 |
| Cow Liver 400 μM EGCG | 132 | 0.3 | 84 | 78 | 386 | 16 |

**Table S1.** Results obtained from the DLS analysis of SAA1 fibril samples derived from the cat spleen, chicken liver, and cow liver. The samples were incubated at 37 °C for 46 hours in 1% DMSO, 200 μM EGCG, or 400 μM EGCG.

**Analysis of EGCG by transmission electron microscopy (TEM).** To confirm that EGCG does not spontaneously form fibrils or other structures, EGCG was prepared at a concentration of 400 µM in 10 mM PBS (pH 7.4) and incubated at 37 °C for seven days. Two samples were prepared and analyzed under the TEM microscope. As shown in **Figure S3**, the EGCG did not form fibrillar or 100 nm vesicular structures under these conditions.


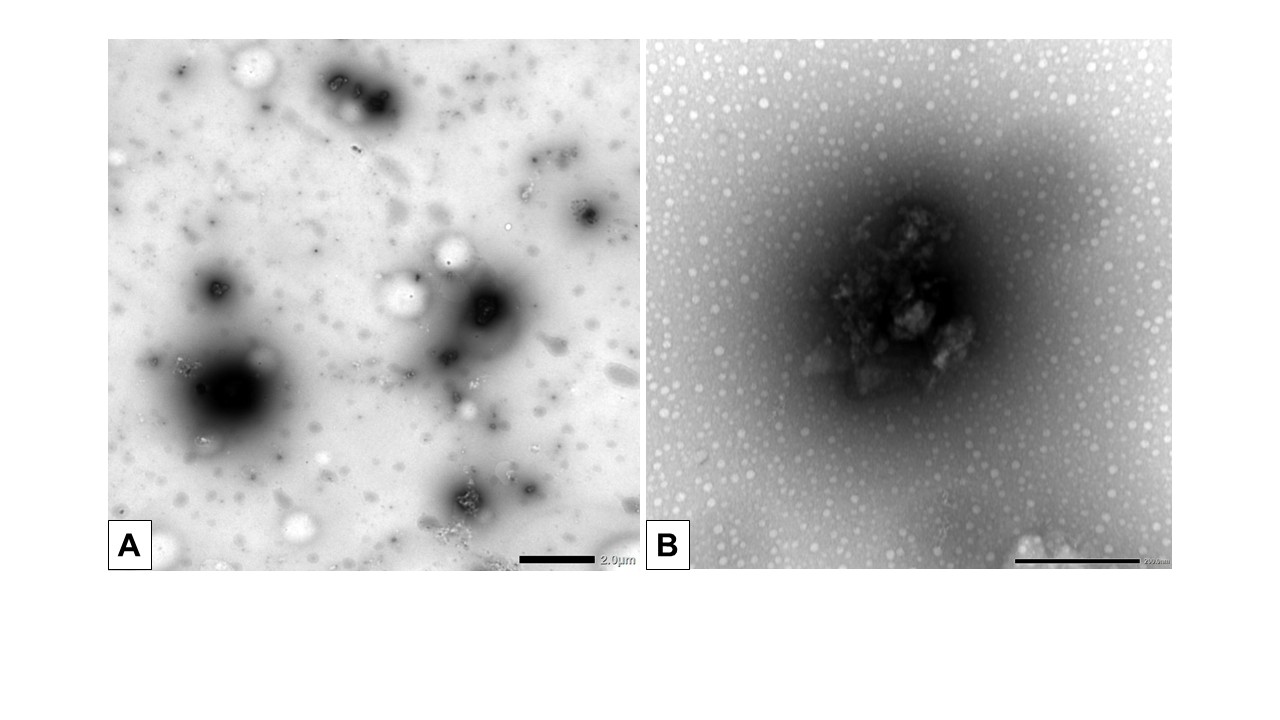
**Figure S3.** Transmission electron microscopy (TEM) imaging of EGCG in the absence of protein at a concentration of 400 µM after incubation in 10 mM PBS (pH 7.4) at 37 °C for seven days. **A)** TEM image of EGCG at 2500x magnification. Sale bar 2 µm. **B)** TEM image of EGCG at 40k magnification. Scale bar 200 nm.

**Examples of area measurements of accumulation of fibrils with ImageJ.** Surface area measurements of accumulation of fibrils were performed with ImageJ for each sample treated with 1% DMSO or 400 µM of EGCG (no centrifugation). Histograms representing the average and SEM of the areas populated by fibrils for each condition are presented in the main manuscript in **Figure 5**. Examples of area measurements with ImageJ are presented in **Figure S4-S6**.


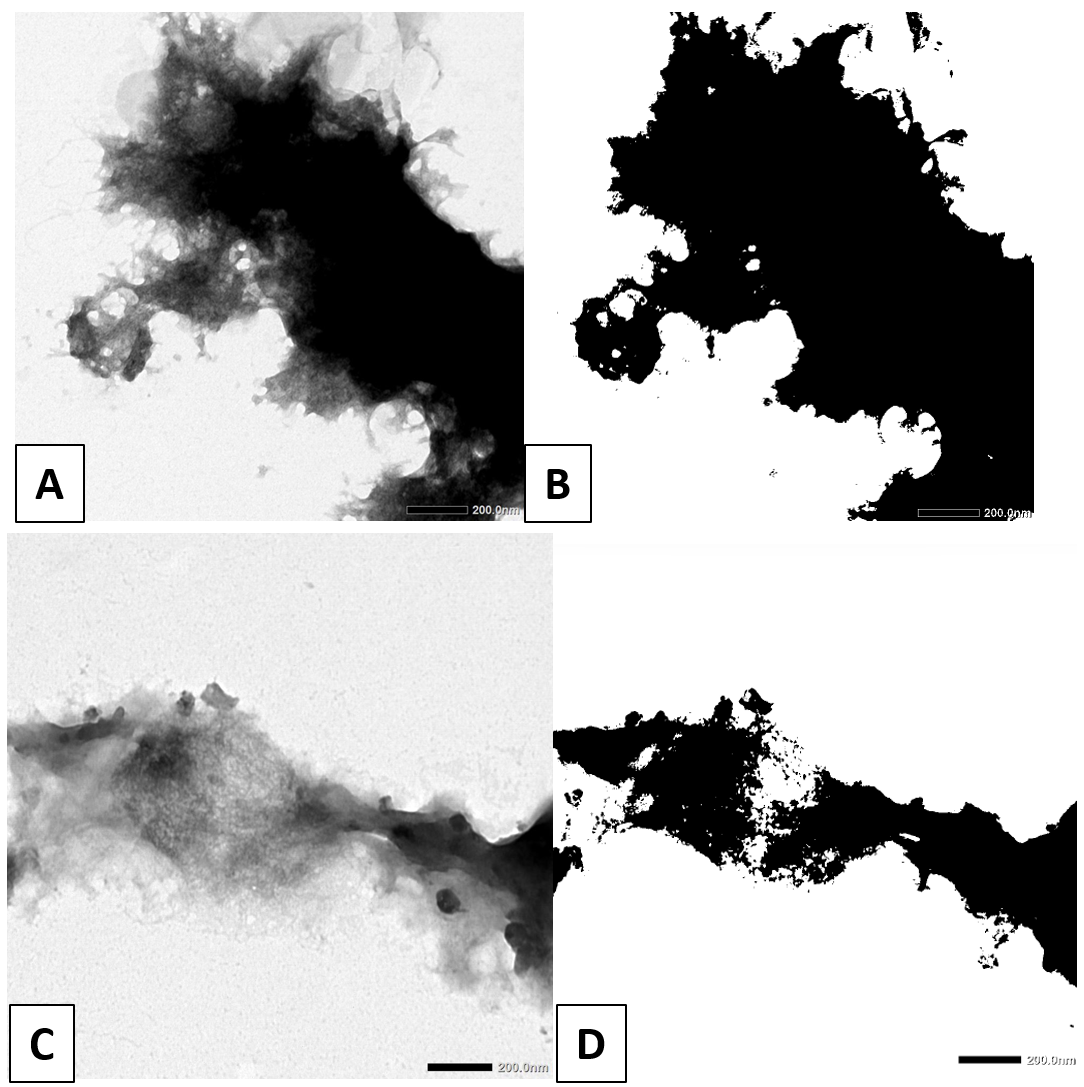
**Figure S4**. Examples of a compact agglomeration of fibrils from the cat spleen surface area measured with ImageJ. **A)** Photomicrograph of the dense agglomerated fibrils incubated with 1% DMSO (20K). **B)** Area captured by ImageJ from the compact fibrils incubated with 1% DMSO (Figure S4A). **C)** Photomicrograph of the less dense accumulation of fibrils resulting from the incubation with 400 µM EGCG (20K). **D)** Area captured by ImageJ from the fibrils incubated with 400 µM EGCG (Figure S4C). Scale bar 200 nm.


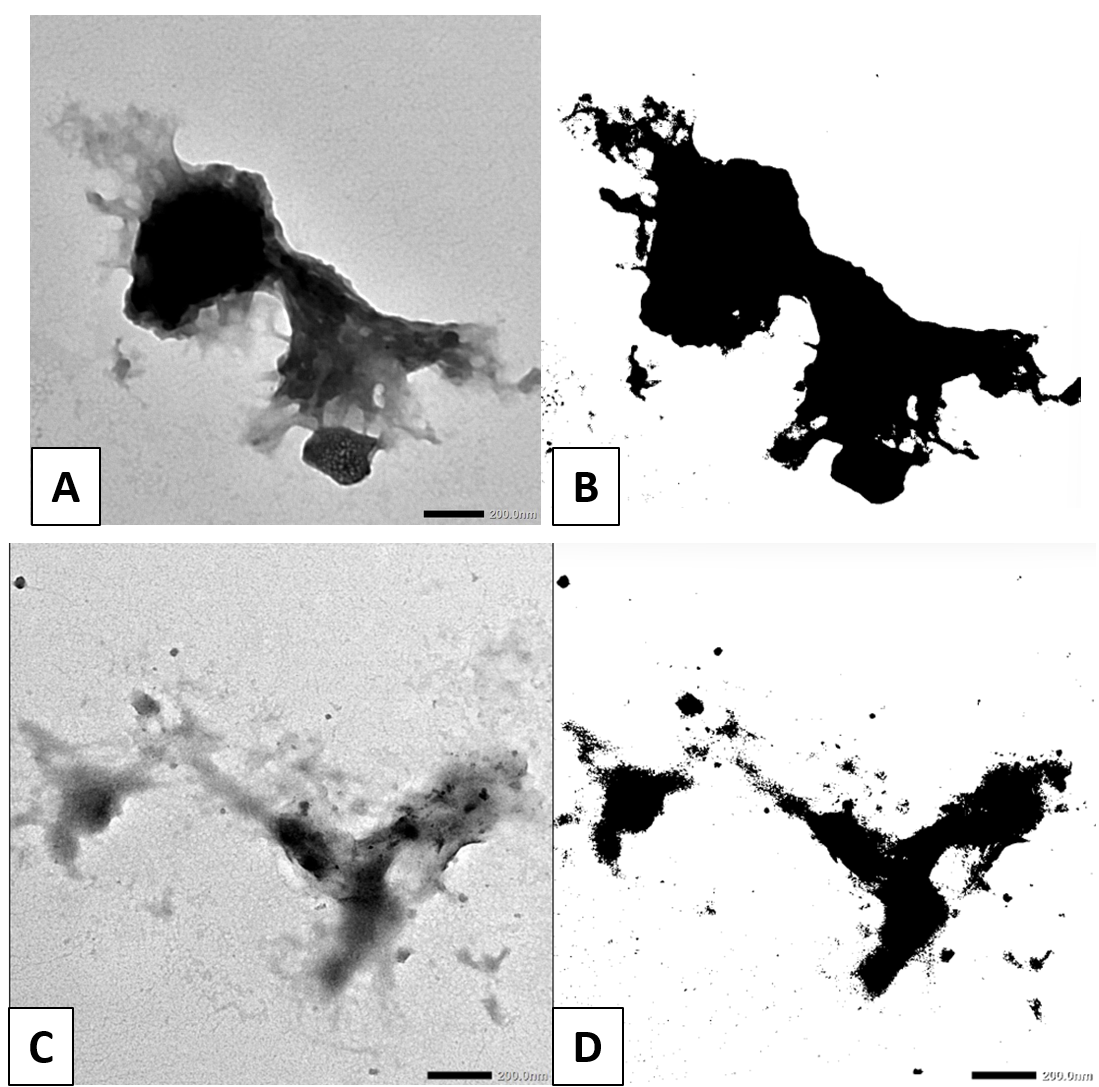
**Figure S5**. Examples of a compact agglomeration of fibrils from the chicken liver surface area measured with ImageJ. **A)** Photomicrograph of the dense agglomerated fibrils incubated with 1% DMSO (20K). **B)** Area captured by ImageJ from the compact fibrils incubated with 1% DMSO (Figure S5A). **C)** Photomicrograph of the less dense accumulation of fibrils resulting from the incubation with 400 µM EGCG (20K). **D)** Area captured by ImageJ from the fibrils incubated with 400 µM EGCG (Figure S5C). Scale bar 200 nm.


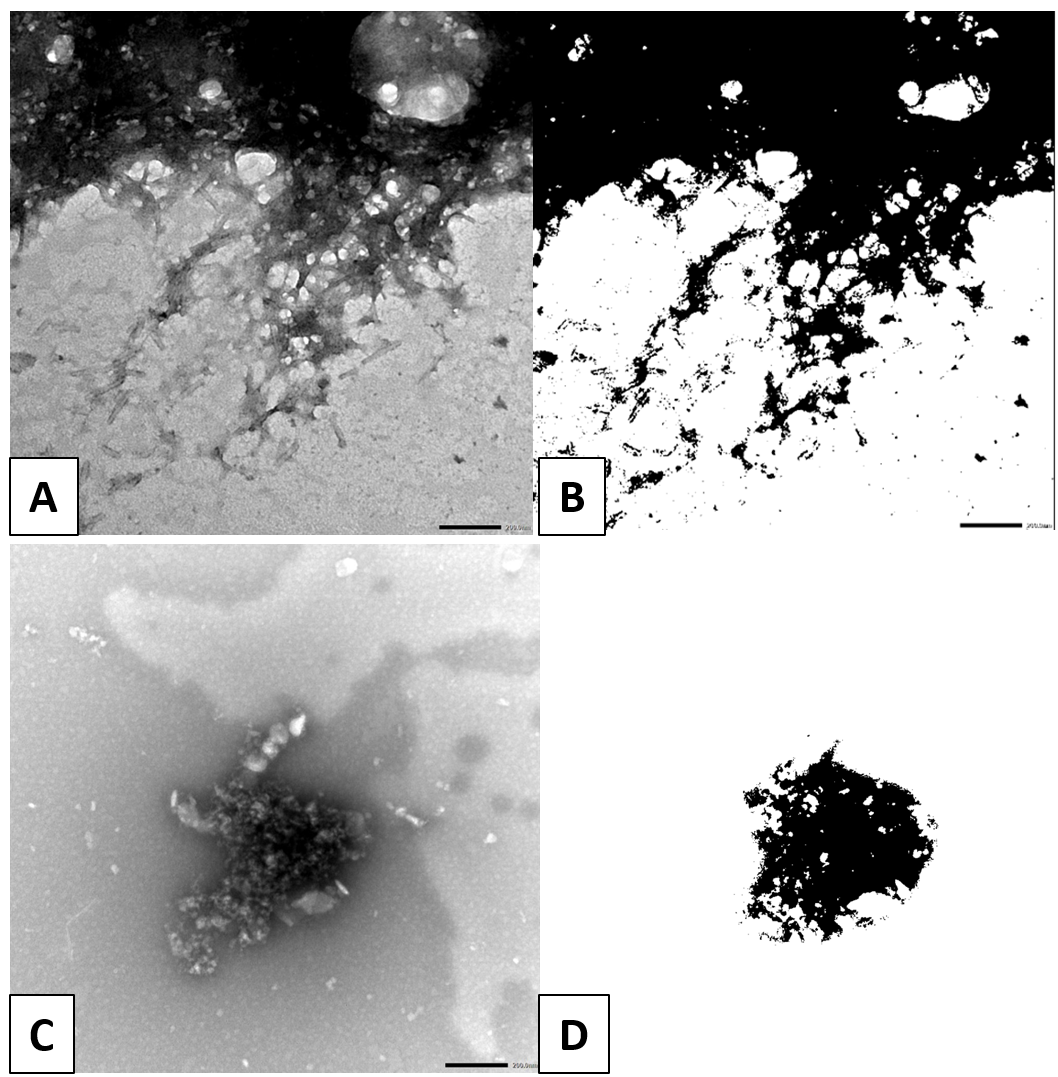
**Figure S6**. Examples of a compact agglomeration of fibrils from the cow liver surface area measured with ImageJ. **A)** Photomicrograph of the dense agglomerated fibrils incubated with 1% DMSO (20K). **B)** Area captured by ImageJ from the compact fibrils incubated with 1% DMSO (Figure S6A). **C)** Photomicrograph of the accumulation of non-fibrillar or not well-defined materials resulting from the incubation with 400 µM EGCG (20K). **D)** Area captured by ImageJ from the resulting structures obtained with 400 µM EGCG (Figure S6C). Scale bar 200 nm.

**Analysis of amyloid plaques by TEM**. To assess the structural changes in amyloid plaques isolated from Alzheimer’s disease brain tissue following EGCG treatment, additional TEM images were obtained. The plaques were incubated in either 0.25% DMSO or 50 µM EGCG in 10 mM PBS (pH 7.4) at 37 °C for five days. As shown in **Figure S7**, the plaques treated with EGCG exhibited non-fibrillar or not-well defined morphology compared to the dense, aggregated fibrillar structures observed in the DMSO-treated control.


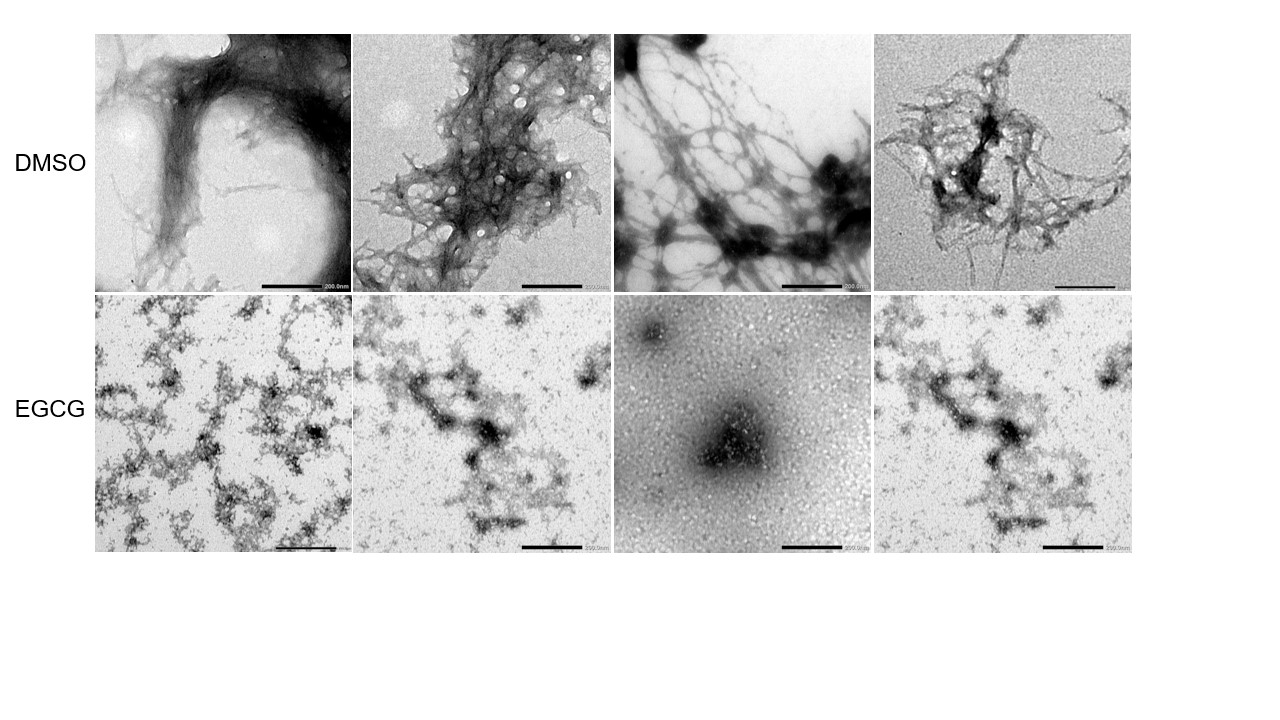


**Figure S7.** Additional TEM images of amyloid plaques isolated from an Alzheimer’s disease brain after incubation with 0.25% DMSO or 50 µM EGCG in 10 mM PBS (pH 7.4) at 37 °C for five days. The samples were not centrifuged prior to grid preparation. The extraction consisted of 0.5 ± 0.1 mg/mL of proteins. The amyloid plaques isolated from the Alzheimer’s disease brain demonstrated dense accumulation of fibrils. Treatment with 50 µM EGCG resulted in non-fibrillar or less defined structures. Scale bar 200 nm.
